# Reducing Healthcare Employees’ Burnout through Ethical Leadership: The Role of Altruism and Motivation

**DOI:** 10.3390/ijerph192013102

**Published:** 2022-10-12

**Authors:** Yushan Wu, Qinghua Fu, Sher Akbar, Sarminah Samad, Ubaldo Comite, Mirela Bucurean, Alina Badulescu

**Affiliations:** 1 School of Economics and Management, Wuhan University, Wuhan 430072, China; 2 Department of Business Administration, Moutai Institute, Zunyi 563000, China; 3 Department of Management Sciences, COMSATS University Islamabad, Islamabad 45550, Pakistan; 4 Department of Business Administration, College of Business and Administration, Princess Nourah bint Abdulrahman University, Riyadh 11671, Saudi Arabia; 5 Department of Business Sciences, University Giustino Fortunato, 82100 Benevento, Italy; 6 Department of Management-Marketing, Faculty of Economic Sciences, University of Oradea, 410087 Oradea, Romania; 7 Department of Economics and Business, Faculty of Economic Sciences, University of Oradea, 410087 Oradea, Romania

**Keywords:** burnout, mental health, healthcare management, altruism, intrinsic motivation

## Abstract

Globally, employee burnout (EBO) is a black swan in healthcare management. Previous organizational management literature shows that EBO was often misunderstood by assuming it as a personal issue. However, the new definition by the World Health Organization (WHO) clearly indicates that EBO is an occupational phenomenon that places responsibility on organizations to manage it. Although recent evidence suggests ethical leadership (ELP) style may be important to mitigate EBO, shockingly, such relationships were not tested in healthcare systems, especially in low- and middle-income countries. Filling this knowledge gap in the existing body of knowledge, this study aimed to investigate the ELP–EBO relationship. To explain the underlying mechanism of how ELP reduces EBO, this study included two psychological factors as a mediator and a moderator: altruism (AL) and intrinsic motivation (IM). The data were obtained from hospital employees via a self-administered questionnaire (*n* = 289, paper-pencil method). A hypothetical framework was designed and tested for empirical validation through structural equation modeling (SEM). Empirical evidence confirmed that ELP reduces the risk of burnout among hospital employees, and AL mediates this relationship. The results also confirmed the conditional indirect role of IM in the above proposed mediated relationship. This study’s outcomes can help hospital administration deal with EBO’s epidemic in an ELP framework. Other, different implications have also been discussed in detail.

## 1. Introduction

Employee burnout (EBO) has been recognized as one of the critical issues in the organizational management literature [1,2]. As a pressing issue, the literature identifies many organizational disruptions associated with EBO [3]. From a financial aspect, it is estimated that EBO makes a dent of billions of USD (more than 300) in the global economy every year, which is larger than the combined profits of the world’s top ten leading businesses, including Apple and Alphabet [4]. GALLUP data on EBO indicate that around 76% of the workforce felt that they were burned-out sometimes. However, around 30% believed they were burned-out often [5]. In its latest definition of EBO, the World Health Organization (WHO) describes EBO as a workplace syndrome associated with unmanaged stress [6]. The literature identifies several factors that give rise to EBO in an organizational context, for instance, stressful working atmosphere, work overload, work–life imbalance, and the blurred nature of a job [7]. Although the negative consequences of EBO have remained a topic of discussion among academicians and practitioners, the demanding working conditions in most professions have perhaps become the common norms, thereby raising the EBO [8]. When EBO increases, an organization’s social fabric is lost, bringing several disruptions.

Though the plight of EBO may exist in any segment of an economy, the literature indicates that it is a pressing issue in a healthcare context [9,10]. Irregular working hours, increasing workload, traumatic events, and other stressors increase the risk of burnout in this sector [11]. The issue of EBO in a healthcare context has more severe consequences compared to other segments of an economy because, globally, the healthcare sector has been identified as a deficient resource sector [12,13], and this resource deficiency issue is especially more critical in many lower- and middle-income countries compared to high-income countries. Therefore, considering the financial costs of EBO, it is worthwhile to reduce EBO in this sector from a developing country standpoint.

A critical issue related to EBO exists with an organizational interpretation of burnout. That is, most of the time, organizations recognize burnout as a personal issue. Rothstein [14] believed that assuming burnout as a personal issue is quite misleading because, in reality, EBO relates more to an organization than a person. A Harvard professor indicated that labeling burnout as a personal issue is wrong because applying personal and band-aid solutions to this epidemic may not help an organization win the battle [15]. Moreover, the new definition of EBO by the WHO (as stated above) clearly places the responsibility on corporate leaders to deal with burnout effectively [4].

Since the emergence of this new definition by the WHO on EBO, there has been a clear surge in the literature on the relationship between leadership and EBO [16,17,18]. In this regard, several leadership models have been proposed by previous scholars to mitigate EBO in different contexts [19,20]. Among different leadership styles, current evidence suggests ethical leadership (ELP) style can mitigate the risk of EBO in an organization [21]. Indeed, ELP is a leadership strategy in which a leader influences followers through ethically normative conduct and develops interpersonal linkages at different stages [22]. The morality, ethics, and interpersonal connection with the employees are some of the major characteristics of ELP, due to which this leadership style can significantly reduce EBO [23]. Although the effectiveness of this leadership style has been recognized at theoretical and empirical levels, such studies are sparse in healthcare systems, especially in a developing economy framework. Therefore, this study bridges this knowledge gap by examining the association between ELP and EBO in a developing country.

Glavas [24] highlighted the importance of different psychological factors as mediators and moderators to better understand and explain the mechanism of different individual outcomes in different contexts. He further stressed that a specific relationship between predictor and criterion variables could better be explained in the presence of mediators or moderators. This is perhaps why, in many behavioral studies, behavioral scientists proposed different psychological factors as mediators or moderators to understand the underlying mechanism of a specific human behavior. We can refer to the studies by Ahmad et al. [25] and Jones et al. [26]. In this regard, past literature notes the importance of different psychological factors as mediators or moderators to predict EBO in an organizational setting, for example, stress [27], psychological contract breach [28], and role conflict [29]. Consistent with this stream of research, we propose individual values, especially altruism (AL), as a mediator to explain the relationship between ELP and EBO. We propose such a mediating role of personal values based on two specific reasons. First, personal values have been found critical to behavior formation at an individual level [30,31]. However, the value framework of an individual provides only a general guide to influence behavior [32], thereby requiring a specific context (ELP in this study) to guide certain employee outcomes (EBO in this study). This implies that investigating personal values’ indirect influence is more worthwhile than examining their direct impact. Previous scholars have also supported this line of reasoning [33,34]. Second, by definition of AL, the ultimate focus of AL is the wellbeing of others [35]. When applied to this study’s theme, healthcare professionals are motivated to serve humanity; hence, this concern for others may help reduce their burnout. This argument is supported by the recent work by Feldman [36] and Saito et al. [37]. Specifically, as the previous literature indicates that personal values are shaped by different social contexts (ELP, for example), it is logical to assume that ELP can influence the AL of employees, which then mediates this relationship.

The existing literature on organizational management highlights the moderating role of intrinsic motivation (IM) to buffer different employee outcomes. Especially, the literature on positive employee psychology discusses the moderating role of IM, for example, job performance [38] and knowledge sharing [39]. Even leadership literature acknowledges the moderating role of IM to buffer different employee outcomes [40]. Conceptually, IM is a personality-related characteristic of a person in which they show motivation for inner satisfaction and not for external rewards [41]. Although empirical evidence suggests the moderating role of IM to buffer different employee outcomes in different contexts, the conditional indirect role of IM between the mediated relationship of ELP and EBO via AL has not yet been tested. This study closes this knowledge gap by examining this proposed relationship.

The rest of this paper is separated into four major sections. The theoretical grounding and relevant literature are discussed in the next section, followed by the methodology, where we provide information about the population, sample, and data collection process. Moreover, the methodology section also provides information about the methods applied. The next segment deals with data analysis to test the hypothesized relationships. The discussion section is the last, where we discuss the results relevant to previous studies with different implications. The potential limitations of this study and the conclusion are also incorporated in this section.

## 2. Literature Review

Based on the conservation of resources (COR) theory, this work suggests that ELP can influence employee outcomes by providing them the necessary resources in times of crisis and to mitigate the threat of EBO. Indeed, Hobfoll [42] presented COR by arguing that employees tend to obtain, develop, and shield different valued resources to protect them against difficult situations. According to Halbesleben et al. [43], there are two types of resources: personal resources and contextual resources, that could help an employee successfully deal with a difficult situation. Although both personal and contextual resources are important to recover from extreme situations in a workplace, contextual resources highlight the critical role of a corporate leader, especially ELP. To this end, the literature argues that an effective leader can serve as a source of contextual resources for the employees [44]. Specifically, a corporate leader such as ELP provides different resources to the employees, for instance, supporting, facilitating, encouraging, and motivating them in difficult times [23]. In addition, ELP maintains interpersonal connections while maintaining fair, honest, and ethical treatment of all employees without prejudice. Consistent with the crux of COR, when employees perceive that their resources are scarce or they have lost some valued resources while fixing an extreme situation, there is a higher chance that this perception will lead them to a burnout situation. In such a situation, ELP provides the employees with the necessary resources, which reduces the threat of resource insufficiency, thereby improving the burnout perception of employees. This is in line with Piccolo and Colquitt [45], who believed that a leader’s role is of profound importance for employees to recover from the risk of resource depletion. Past researchers have also used COR to explain the underlying mechanism of how leaders could be a source of contextual resources for employees to recover from the risk of burnout. The work by Afshan et al. [46] is a case in point.

ELP as an ethical person tends to conform to a comprehensive code of conduct that is based on morality and ethics in a workplace [47]. The moral practice and conduct of an ELP directly influence the morality level of employees. Employees with an enhanced level of morale tend to develop a higher level of mental health, which ultimately provides them added support in difficult times [48]. Being the executor of ethics, ELP infuses ethical principles among employees, which ultimately encourages them not to give up in extreme situations [49]. Conceptually, employees are likely to emulate the ethical role model of their corporate leaders on their own [50]. The principal focus of an ELP is on fairness, respect, interconnectedness with employees, and on ethics. Employees, as the receiver of such benefits and support from an ELP, respond positively and happily go beyond the formal working lines of their duty [51]. Generally, employees working under the leadership of an ethical leader have more access to different valuable psychological resources, including interpersonal trust, respect, cooperation, and flexibility. Past literature suggests that the psychological resources provided by a corporate leader to the employees help them to fight against burnout, which is a “black swan” in healthcare management [52,53]. Additionally, by referring to COR, the supportive environment provided by an ELP in an organization serves as a contextual resource that empowers employees to deal with difficult situations without any fear of resource loss or depletion. Therefore, consistent with previous research on the ELP–EBO relationship [54,55], we propose:

**H.:**  *The manifestation of ELP in a healthcare organization reduces EBO.*

Basically, AL focuses on the wellbeing of others even at a cost or risk to oneself [37]. Some behavioral scholars believe that AL is at the core of the healthcare profession [56]. AL is often assumed to be a selfless virtue of an individual. Nevertheless, evidence suggests that AL should not be assumed only as an advanced version of morality that overturns an individual’s ego-driven urges [36]. Indeed, AL is deeply wired in the human brain and is influenced/shaped by several factors [57], which include, but are not limited to, various social, cultural, and religious factors [58], because there is clear evidence in the available literature that AL at an individual level is shaped by different factors [59,60]. Such evidence has attracted the attention of behavioral scientists to examine whether leadership influences AL in an organization. Fortunately, the existing body of knowledge recognizes the role of corporate leaders to influence/shape the AL of employees [61]. Even the mediating effect of AL in a leadership context was highlighted several times in the available literature [62,63]. Our argument here is that ELP influences the AL of employees because an ELP not only encourages and facilitates the employees in difficult times, but they also show caring concern for employees’ wellbeing. Employees reciprocate the same and develop this caring concern for others on their part too; therefore, they are expected to show altruistic behavior while performing their job. This is in agreement with the current work by Zhu et al. [64].

Interestingly the existing body of knowledge conceptualizes that in a healthcare profession, AL can reduce EBO. For instance, Oakley et al. [65] mentioned that AL keeps the morale of healthcare professionals high, which reduces the risk of EBO. Specifically, Shaw et al. [66] showed that healthcare professionals were encouraged by their AL to serve humanity even at the risk of their personal health (for example, the risk of being infected). Haigh [67] believed that employees in a healthcare profession care for patients and place AL at the core of their professional life for the sake of humanity. Altogether, because AL in healthcare encourages a person to work for the good sake of humanity, altruistic employees are less likely to be burned out. When linked with ELP, the following hypothesis may be proposed:

**H2.**  *The manifestation of ELP in a healthcare organization can positively influence employees’ AL.*

**H3.**  *Employees’ AL in a healthcare organization mediates the relationship between ELP and EBO.*

An intrinsically motivated person is likely to show extra commitment to achieve an objective for their inner satisfaction [68]. Indeed, research shows that a person with IM can even go beyond formal job obligations and puts extra effort into a mission or goal [69]. Moreover, IM encourages an employee in a workplace to accept different challenges enthusiastically [70]. Employees with IM show an enhanced level of energy, self-esteem, and absorption capacity when exposed to difficult situations [71]. Because employees with high IM are more positive in pursuing different organizational goals, they do not feel a resource insufficiency or loss situation while being exposed to uncertain situations. Recently, organizational interest has been increasing in exploring the factors that can increase the IM of employees [72]. Intrinsically motivated employees not only contribute toward organizational excellence, but such employees also face less risk of being burned-out [73].

**Figure 1:**
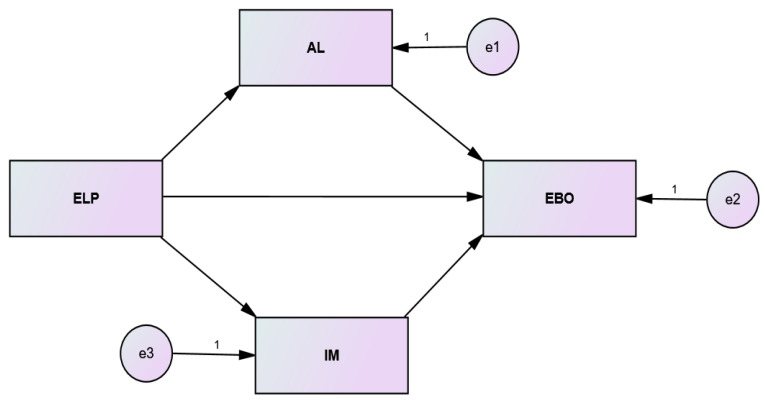
The hypothesized structural model.

The existing body of knowledge indicates that corporate leaders, as a bottom-up management approach, are in a position to enhance the IM of employees [74]. Suzanne [75] indicated that intrinsic motivation could be learned, developed, and taught to an individual. She further emphasized that in an educational context, a teacher who is more connected with the students and gives them more learning opportunities can successfully infuse IM among the students. We argue that the same holds for employees in an organization under the supervision of ELP because an ethical manager is interconnected with the employees, involves them in organizational decision-making, and facilitates them by providing necessary resources. Theoretically, all these processes can be linked to a higher level of IM. The authors such as Yidong and Xinxin [76] and Feng et al. [77] also shared the same viewpoint by indicating that an ELP is a source of IM for employees. Moreover, because ELP shows concern for the wellbeing of employees, such caring concern boosts the morale of employees, thereby increasing IM [78]. Conceptually, it is possible to assume that employees with a higher level of IM, as an antecedent of ELP, can develop a better level of AL. Because an altruistic person shows concern for the welfare of others, and when such persons are intrinsically motivated, it is expected that AL will be stronger, which will, in turn, reduce EBO. Please refer to Figure 1 for hypothesized structural model. Therefore,

**H4.**  *ELP enhances IM of employees in a healthcare organization.*

**H5.**  *IM moderates the mediated relation between ELP and EBO in healthcare via AL, such that the relationship between ELP and AL is expected to be stronger in the presence of IM.*

## 3. Methodology

### 3.1. Unit of Analysis, Sample, and Procedure

In this study, individual employees serving in different hospitals in Lahore city were the unit of analysis. Lahore is the provincial capital of Pakistan’s largest province (in terms of population), Punjab. This city constitutes a multi-million population (around 13 million) [79]. Hospitals in this city (both public and private) have to attend to a bulk of patients daily. Unfortunately, the public health status of Pakistan is not good. The rank and files in the country face different communicable diseases due to different factors, including overpopulated cities, contaminated drinking water, poor sanitation, low health-related education, and inadequate socio-economic conditions. The poor public health statistics contribute to increasing patient traffic in hospitals, especially in large cities. The over-crowded situation in most hospitals, accompanied by poor physician-to-patient and nurse-to-patient ratios, are some of the reasons that have overburdened hospital staff, thereby increasing the risk of EBO. As a lower middle-income country, Pakistan faces a poor health facility situation as Pakistan has been ranked 154th in the list of 195 countries in terms of health facilities [80].

To collect the data for this study, we randomly contacted different hospitals to seek their permission to directly contact the employees. Specifically, six hospitals agreed to facilitate us in the activity of data collection. The sample included healthcare employees from different departments (physicians, para-medical, administration, etc.) and ranks (manager and nonmanager). Particularly, the data collection was completed within two months (February to March 2022).

### 3.2. Data Collection Instrument and Measures

We used a questionnaire (self-administered) to collect the responses from the participants. The items used in this survey to measure this study’s variables (ELP, EBO, AL, and IM) were taken from different reliable sources. Experts’ assessment was also obtained before presenting the data collection instrument to the employees [81,82,83,84]. Specifically, the outlook of the questionnaire was two-fold, consisting of the socio-demographic information and variable-related ratings on a 5-point Likert scale. Regarding the ethics in data collection activity, we confirm that the major principles defined in the Helsinki Declaration had been observed [85,86,87,88].

There were four variables in this study. The predictor variable (ELP) was measured by adapting the famous and reliable scale of Brown et al. [22]. This included a total of 10 items (for instance: “our manager/leader discusses business ethics or values with us” and “our manager/leader sets an example of how to do things the right way in terms of ethics”). EBO was the criterion variable in this study that was measured by adapting the scale of Kristensen et al. [89]. Indeed, this scale included a total of 7 items, including the illustrated items “I feel worn out at the end of the working day” and “I am exhausted in the morning at the thought of another day at work.” The intervening variable (AL) was measured by using a 3-item scale by Dotson et al. [90]. One illustrated item from this scale was “I became a healthcare professional to help others.” Lastly, the interacting variable (IM) was measured with the help of 5 items that were taken from the study of Tierney et al. [91]. An illustrated item included “I enjoy finding solutions to complex problems.” All variables showed significant inter-item consistency (α) values (ELP = 0.89, EBO = 0.91, AL = 0.75, and IM = 0.88).

It is important to mention here that we dealt with social desirability and common method variance (CMV) by employing a multi-wave data collection strategy. Specifically, the data were collected in three separate waves (with an interval of two weeks between each wave).

### 3.3. Sample Size and Data Cleaning

We used an a priori sample size calculator to decide on the minimum sample size for this study. Developed by Dniel [92], this calculator estimates a study-specific sample size. Particularly, this calculator decides on the number of samples for a particular study by considering different decision criteria, including the number of observed variables (25 in this study) and unobserved variables (5 in this study). The study-specific estimation of this application, accompanied by other, different benefits, are some of the important reasons making this calculator a contemporary tool for sample size estimation. Indeed, Memon et al. [93] mentioned that this sample size application is equally important for any structural equation modeling (SEM) technique (CB-SEM and PLS-SEM). Based on specific inputs, the a priori calculator estimated that the minimum recommended sample size for this study should be 229.

Knowing the fact that survey research does not produce a 100% response rate, we distributed 450 questionnaires among the employees of different hospitals. After three waves of data collection, we received back 328 responses. However, after data cleaning (deleting outliers and responses with missing information), we included 289 valid responses in the final dataset. We employed a Mahalanobis test in AMOS to detect outliers (9 cases). For more detail, we refer to Table 1 and Table 2. The sociodemographic stats revealed that both male and female employees responded to this data collection activity (62% were male). Similarly, the ages of maximum employees were between 18 and 45 years (around 86%). The experience varied between 1 and 10 (89%).

## 4. Results

### 4.1. Common Method Variance

Though we collected the data in multi-waves, we still performed a robust test to detect CMV by performing a common latent factor (CLF) test. This analysis was performed in AMOS software. Particularly, we developed two measurement models (hypothesized vs. alternate). The hypothesized model did not include any CLF; in contrast, the alternate model included a CLF. This CLF was introduced to affect all observed variables directly. The standardized factor loadings of both models were observed and compared. It was realized that both models did not vary significantly (>0.2), indicating that a CLF did not produce any significant difference. Thus, the potential issue of CMV did not exist in this study, and even if it existed, it was not critical.

### 4.2. Reliability and Validity

To establish the validity and reliability of this study’s variables, we calculated the average variance extracted (AVE) for all variables individually. For such calculations, first, we checked if the factor loadings in all cases were appropriate (not <0.5). Table 3 represents a snapshot of different statistical outcomes for the convenience of the readers. In light of the outputs given in Table 3, it can be seen that the factor loadings were significant and positive in all cases. Based on these values, we were able to estimate AVEs for all variables. For such calculations, we used Equation (1):

(1) $$\mathrm{AVE}=\frac{\sum_{\dot{i}=1}^{k}\lambda_{i}^{2}}{\sum_{\dot{i}=1}^{k}\lambda_{i}^{2}+\sum_{i=1}^{k}.var\left(\varepsilon i\right)}$$

The AVEs were more than the standard cut-off value (>0.5) for all variables. The values varied from 0.54 (AL) to 0.66 (IM). Usually, AVE > 0.5 indicates that the convergent validity of a variable is significant. This is why we infer that the validity of all variables was significant.

Similarly, we calculated reliability, especially composite reliability, by employing the formula shown in Equation (2).

(2) $$\mathrm{Composite}\;\mathrm{reliability}=\left(\left(\sum^{\;}\lambda i\right)2\right)/\left(\sum^{\;}\lambda i\right)2+\sum^{\;}var\left(\varepsilon i\right)$$

The output confirmed that the reliability was significant (>0.7) for all variables. Specifically, the values varied from 0.78 (AL) to 0.93 (ELP). These results indicated that the reliability condition for all variables was fulfilled, giving a go-ahead to carry the data analysis to the next level.

### 4.3. Model Fitness

To assess whether this study’s originally hypothesized model (four-factor) fits well with the statistical data, we developed different measurement models with different compositions (for more detail, please see Table 4). The prime objective of carrying out such an analysis was to make a comparison to decide if the original baseline model (model-1) was superior to the alternate models (model 2, 3, and 4) [94,95]. Particularly, three alternate models were developed, which included a 1-factor model (ELP+EBO+AL+IM), a 2-factor model (ELP+IM and EBO+AL), and a 3-factor model (ELP+IM, EBO, and AL). To make a decision, different model fit indices were observed, which included goodness of fit index→GFI, Tucker Lewis index→TLI, incremental fit index→IFI, comparative fit index→CFI, root-mean-square error of approximation→RMSEA, and chi-square→*χ*^2^ values, which were observed against their standard values. The comparison revealed that a 1-factor model was poorly fit to the data, whereas a 2-factor and a 3-factor model produced better results (especially in a 3-factor model). However, the originally hypothesized model outperformed all alternate models (GFI = 0.95, TLI = 0.95, IFI = 0.96, CFI = 0.95, RMSEA = 0.05, and *χ*^2^*/df* = 2.29). Such a comparison helped us to empirically establish that model-1 fits well to the data.

### 4.4. Correlations

A correlation analysis was carried out to determine the nature of the relationship among different variable pairs. The preliminary analysis revealed that some pairs had a positive correlation, whereas others produced a negative one. However, all correlations were significant (either positive or negative). A case in point may be seen in Table 5 between ELP and EBO, which shows a negative but significant correlation (ELP<=>EBO = −0.39). Similarly, the correlation between ELP and AL was positive (ELP<=>AL = 0.46). The correlation analysis provided preliminary support to the theoretical statements of hypotheses; for example, H1 claimed that ELP was negatively related to EBO, and the correlation value was also negative. Moreover, no variable pair produced an extreme value (>0.8), which shows that multicollinearity did not occur in this analysis. We also reported discriminant validity results in Table 5 (bold values), which showed that the items of one variable did not match with the items of any other variable.

### 4.5. Hypotheses Testing

We tested the hypothesized relationships by pursuing structural equation modeling (SEM). SPSS-17 and AMOS software (version 23) were considered for SEM analysis. In particular, we used the PROCESS macro developed by Hayes [96]. This macro helped us calculate different hypothesized relationships through different equations installed in AMOS (by enabling user-defined syntax). It is important to mention here is that the predictor variable (ELP) and moderating variable (IM) were mean-centered prior to performing the structural analysis [87,97]. Further, an interaction term (ELP_x_IM) was also developed to see the conditional indirect effect. Model-7 of the PROCESS macro provided the baseline to conduct this analysis in AMOS [98]. To see the mediating and moderating effects, a larger bootstrapping sample of 5000 [99] was considered in AMOS. A snapshot of different hypothesized relationships is presented in Table 6 for the readers. As per the results, H1, H2, and H4 were found significant as these hypotheses were supported by empirical evidence (−0.41, 0.44, 0.47, *p* < 0.5 with non-zero confidence interval values). The bootstrapping results confirmed the mediating effect of AL between ELP and EBO (ELP→AL→EBO = −0.34, *p* < 0.5). This confirms the theoretical statement of H3, thereby indicating that H3 was significant.

We tested the conditional effect of IM between ELP and AL at three different points, including at the mean and ± 1 SD of the mean. The results confirmed the conditional effect of IM between ELP and AL. Lastly, we reported the conditional indirect effect of AL between the mediated relationship of ELP and EBO via AL, which was significant (−0.25, *p* < 0.5, with nonzero confidence intervals). This implies that AL buffers the mediated relationship between ELP and EBO, confirming that H5 was also significant.

## 5. Discussion

This study was carried out to determine the effect of ELP on EBO in the healthcare sector of Pakistan. The empirical findings confirmed our theoretical assumption that the manifestation of an ELP in a hospital can help employees to reduce the risk of being burned-out (beta value = −0.41). The morality, ethics, and interpersonal connection with the employees are some of the major characteristics of ELP that serve as the base of additional valuable resources to strengthen employees against the risk of burnout. Being an ethical manager, an ELP tends to develop different codes of ethical conduct that are based on morality and ethics. The ethical conduct of an ELP boosts the morality level of hospital employees. From this standpoint, employees with an enhanced level of morale, as an antecedent of ELP, are likely to have an improved level of mental health, which provides them with an added strength in difficult situations to lessen the risk of burnout. In addition, an ELP gives employees more access to various psychological and physical resources. Employees as the receiver of such resources are expected to overcome the fear of resource bleeding or resource loss when facing a difficult situation. In line with COR, the supportive environment provided by an ELP in an organization serves as a contextual resource that empowers employees to deal with difficult situations without any fear of resource loss. All these processes make the employees more resource-equipped, thereby reducing EBO. This finding is consistent with previous research [54,55].

Additionally, our results highlight the profound importance of personal values, especially AL, to explain the underlying mechanism of how and why the manifestation of ELP in a hospital reduces EBO. To this end, the ultimate focus of AL is the wellbeing of others. Especially in healthcare, AL is of prime importance because past evidence suggests that healthcare professionals are motivated to serve humanity, and hence, this concern for others increases their commitment to their profession, which helps employees in reducing their burnout. AL serves as a personal resource that limits the risk of burnout among hospital employees. Consistent with previous research, our findings revealed that ELP produces a positive impact on the AL (beta value = 0.44) of employees, which then mediates the ELP–EBO relationship (beta value = −0.34)

Indeed, AL is influenced/shaped by several social, cultural, and religious factors. Similarly, in an organizational setting, leadership influences AL. This finding is consistent with Sagnak and Kuruöz [61]. Specifically, ELP facilitates and supports employees at one end, and they also show caring concern for employees’ wellbeing. Employees tend to reciprocate the same by developing this caring concern for others on their part too, and thereby, they are likely to show more altruistic behavior. Past research also confirms this finding of our research [64]. Hence, we confirm the mediating effect of AL between ELP and EBO.

Lastly, our results indicate that IM moderates the mediated relationship between ELP and EBO via AL. Indeed, the inclusion of IM into the theoretical model of this study confirmed that IM produces a buffering effect on the above-mediated relationship. Consistent with previous research, we confirm that IM encourages an employee in a workplace to accept different challenges enthusiastically. Employees with higher enthusiasm are more energetic and have better absorption capacity in difficult situations. This is in line with previous studies, too [70,71]. Intrinsically motivated individuals are more positive and do not feel a resource insufficiency or loss situation while being exposed to uncertain situations. We confirm that an ELP, as a bottom-up management approach, is in a position to enhance the IM of hospital employees, which then buffers between ELP and AL to reduce EBO. Therefore, the conditional indirect effect of AL was confirmed in this study.

### 5.1. Theoretical Contribution

This study contributes to the existing body of knowledge in three ways. First, our study extends the discussion on EBO in a leadership framework, especially by highlighting the role of an ELP. Although leadership scholars have highlighted the role of different leadership styles in reducing EBO in different contexts [23,100], the role of ELP in mitigating the risk of burnout in the healthcare sector, especially in low- and middle-income countries (for example, Pakistan), remained under-explored. We feel this study’s contribution to reducing burnout among hospital employees from a developing country context is important because employees face more difficult social and structural situations than employees in developed countries because developing countries do not have sufficient resources and infrastructures. Therefore, this study contributes significantly to the existing literature by highlighting that ELP in a certain hospital in a developing country can help employees to reduce burnout risk.

Second, this study is an important contribution to the existing body of knowledge related to human psychology and organizational management because this study proposed a robust model to explain the underlying mechanism of EBO in a leadership framework. The reason we feel this model is a robust model lies behind the fact that human psychology is complex to understand and is shaped by different organizational and personal factors accompanied by personality characteristics. From this perspective, the theoretical model of this study is unique because it not only considered organizational factors (ELP) but also realized the importance of personal and personality-related factors (AL and IM). Including such psychological factors helps to understand the underlying mechanism of how an ELP reduces EBO by influencing the AL and IM of employees. Specifically, our study extends the theoretical model by Tu and Lu [101]. These authors did a decent job by highlighting the conditional indirect role of IM in an ELP framework to improve employee extra-role performance through the mediating effect of efficacy (a personal factor). However, these authors did miss highlighting the importance of ELP to improve a specific employee outcome (burnout, for example). Our research also extends the discussion by Okpozo, A.Z., et. al. [102], who decently highlighted that AL is at the core of healthcare employees, but the missing factor from Feldman’s discussion was the intervention of an effective leadership style.

Third, our study also makes a significant contribution to the existing literature on employee psychology and organizational management literature by interpreting the new definition of burnout by the WHO by linking it to leadership. Because the new definition of EBO clearly indicated that burnout is an organizational phenomenon, not personal, it was important to discuss how leadership (organizational factor) could reduce EBO, especially in a healthcare system. Specifically, this study highlights that assuming burnout as a personal issue is misleading because applying personal and band-aid solutions to this epidemic will not help a hospital to win the battle against the EBO, which is a “black swan” in the healthcare system.

### 5.2. Practical Contribution

On a practical landscape, this study offers significant insights to the management of hospitals to prepare for the “black swan” that is EBO in this sector. Considering organizational disruptions and different negativities associated with EBO in the healthcare system, we recommend the management of a certain hospital to promote an ethical style of leadership as an effective organizational management strategy to mitigate EBO. This finding has profound importance for the healthcare sector because burnout has been identified as an epidemic in this sector worldwide. This sector’s demanding and irregular working conditions put pressure on employees and create challenges to fulfill their job responsibilities. In this regard, an ELP can significantly reduce employees’ burnout risk by encouraging, motivating, and helping them in difficult times. The ethical and supporting conduct of ELP keeps employee morale high, thereby providing them additional resources to fight against EBO.

Another important point that our research offers to the healthcare sector is the financial cost associated with EBO. Given that, globally, healthcare systems face resource insufficiency, the high cost of burnout needs to be mitigated, for which an ethical leadership framework is a way forward. More importantly, from the standpoint of lower- and middle-income countries, most of these countries’ healthcare systems face insufficient resources, including financial, human, institutional, and infrastructure-related resources. The financial leakage in the form of EBO creates a further dent to the weak health systems in developing countries. Therefore, if EBO in this sector is managed effectively, it will improve the resource efficiency at one end and improve the mental health of employees at the other end, which is very important for a better healthcare service delivery. All in all, an ethical leadership framework has clear implications for this sector to reduce EBO and for effective organizational management.

### 5.3. Limitations and Future Suggestions

Although this study offers different implications on theoretical and practical landscapes, it still faces some potential limitations that we consider important to highlight, along with different suggestions. First, this study collected the data only from a single city (Lahore) in Pakistan. Though the city constitutes a very large number of hospitals (both public and private), we feel the geographic concentration of this study may limit its generalizability. Therefore, we recommend including more cities from other provinces (Karachi, for example). Sample representativeness is another potential issue, although the data were collected from healthcare employees. However, due to different policy reasons and security parameters, most hospitals were only kind to let us enter their premises for data collection, but no hospital shared any statistics on hospital employees, departments, etc. Due to this difficulty, deciding on sample representativeness was not possible. In the future, we suggest incorporating this limitation by collecting such statistics on employees (if possible). Another potential issue with this study was the nonprobability sampling technique, which limits the significance of causal relationships. Therefore, in future studies, we suggest pursuing a probability sampling method to have a better causality claim. Further, as culture may be important in leadership studies, we feel that, in similar cultures (India, for example), our study may reflect similar results; however, a due consideration is requested before interpreting our results in different cultures (Western culture, for example). Similarly, as organizational factors such as environment, organizational culture, and ownership structure influence leadership style, we suggest in future studies that such factors may also be considered. Similarly, there may be a two-way relationship between employees and organizational resilience [103], which may be explored in future surveys. In a like manner, it may be worthwhile to determine in future studies how cognitive, behavioral, and contextual dimensions of organization resilience, at different levels in an organization [104], may be helpful in dealing with EBO in a leadership framework. Lastly, as suggested by Cruickshank [105], it may also be investigated in future studies how organizational resilience strategies may be helpful in dealing with crisis situations (for example, EBO) by cultivating different organizational interventions, for example, an effective leadership style.

## 6. Conclusions

For better organizational management and to deal with the epidemic of burnout in the healthcare system, ELP may be an effective bottom-up approach. Moreover, the new definition of EBO clearly places the responsibility on organizations to take effective measures to mitigate the risk of burnout on the part of employees. We suggest a hospital management to promote an ethical leadership style of management at all levels. We propose to design and arrange different seminars and training programs for hospital managers with a special focus on converting them into ethical managers. We further suggest that such training programs should be closely aligned to improve the IM and AL of employees as antecedents of leadership. When a leader is able to improve the AL and IM of employees (which is quite possible for an ELP), the energy and morale level of employees are improved, thereby improving their mental health and reducing the risk of being burned-out. Additionally, a leader with ethical orientation also improves the resilience level of employees through their ethical conduct in a workplace. Resilient employees, due to the ethical conduct of their leader, show an extra level of energy to resist burnout. All in all, if burnout is the “black swan” in healthcare, ethical leadership is the way forward to prepare for this “black swan.”

## Figures and Tables

**Table 1:** Data cleaning stats.

	Initial Sample	Received	Not-Received	Deleted	Outliers	Final Response
	450	328	122	39	9	289
Percentage	-	72.89	27.11	11.89	2.31	64.22

**Table 2:** Observations identified as outliers.

Response Number	Mahalanobis D-Squared	p1	p2
167	22.473	0.001	0.000
221	22.473	0.001	0.000
2	20.492	0.002	0.000
43	18.371	0.005	0.000
4	16.987	0.009	0.000
170	16.987	0.009	0.000
186	15.480	0.017	0.000
240	15.480	0.017	0.000
25	14.320	0.026	0.000

**Table 3:** Validity and reliability.

	Λ	λ^2^	E-Variance
ELP			
	0.72	0.52	0.48
	0.76	0.58	0.42
	0.72	0.52	0.48
AVE = 0.59	0.85	0.72	0.28
CR = 0.93	0.79	0.62	0.38
∑λ^2^ = 5.85	0.70	0.49	0.51
Items = 10	0.73	0.53	0.47
	0.88	0.77	0.23
	0.74	0.55	0.45
	0.74	0.55	0.45
EBO			
	0.69	0.48	0.52
	0.72	0.52	0.48
AVE = 0.55	0.77	0.59	0.41
CR = 0.90	0.81	0.66	0.34
∑λ^2^ = 3.86	0.73	0.53	0.47
Items = 7	0.71	0.50	0.50
	0.76	0.58	0.42
AL			
AVE = 0.54	0.70	0.49	0.51
CR = 0.78	0.74	0.55	0.45
∑λ^2^ = 1.63	0.77	0.59	0.41
Items = 3	-	-	-
IM			
	0.77	0.59	0.41
AVE = 0.66	0.81	0.66	0.34
CR = 0.91	0.84	0.71	0.29
∑λ^2^ = 3.32	0.88	0.77	0.23
Items = 5	0.77	0.59	0.41

Notes: λ = Item loadings, CR = composite reliability, ∑λ^2^ = sum of square of item loadings, E-Variance = error variance.

**Table 4:** Model fit comparison, alternate vs. hypothesized models.

Model	Composition	*χ*^2^*/df* (<3)	GFI (>0.9)	TLI (>0.9)	IFI (>0.9)	CFI (>0.9)	RMSEA (<0.08)	Δ*χ*^2^/*df* -
1	(Original) ELP, EBO, AL, IM	2.29	0.95	0.95	0.96	0.95	0.05	_
2	(Alternate 3-factor) ELP+IM, EBO, AL	5.83	0.72	0.74	0.75	0.74	0.07	3.54
3	(Alternate 2-factor) ELP+IM, EBO+AL	6.77	0.59	0.60	0.60	0.61	0.09	0.94
4	(Alternate 1-factor) ELP+EBO+AL+IM	8.96	0.50	0.56	0.55	0.55	0.22	2.19

**Table 5:** Correlations and discriminant validity.

Construct	ELP	EBO	AL	IM
ELP	**0.73**	−0.39	0.46	0.49
EBO	(2.59, 0.42)	**0.75**	−0.36	−0.40
AL		(3.16, 0.54)	**0.74**	0.52
IM			(3.06, 0.51)	**0.77**
				(2.77, 0.43)

Notes: Diagonals = Mean and standard deviation, bold = discriminant validity values, *p* < 0.001.

**Table 6:** Direct, indirect, and conditional effects.

Hypotheses	Estimates (SE)	*t/z*	*p*-Value	CI
(ELP→EBO)	−0.41(0.069)	−5.94	****	−0.56, −0.33
(ELP→AL)	0.44(0.060)	7.33	****	0.29, 0.54
(ELP→IM)	0.47(0.057)	8.24	****	0.36, 0.62
Indirect effect (ELP→AL→EBO)	−0.34(0.041)	−8.29	****	−0.47, −0.36
Conditional effect of IM between ELP and AL				
−1SD	0.044(0.016)	-	-	0.27, 0.49
At mean	0.042(0.013)	-	-	0.30, 0.54
+1SD	0.039(0.017)	-	-	0.27, 0.42
Conditional indirect effect of IM between ELP→AL→EBO	−0.25(0.033)	−7.58	****	−0.31, −0.19

Notes: CI = 95% confidence interval with lower and upper limits. ****, shows the level of confidence at 99 percent.

## Data Availability

Data will be available on demand.
